# *Euphorbia hypericifolia* Attenuates Citrinin-Induced Oxidative Stress and Maintains Tight Junction Integrity in Porcine Intestinal Epithelial Cells

**DOI:** 10.3390/ijms26167773

**Published:** 2025-08-12

**Authors:** Seung Joon Lim, Sangsu Shin, Tae Hyun Kim, Sang In Lee

**Affiliations:** 1 Department of Animal Science and Biotechnology, Kyungpook National University, Sangju 37224, Republic of Korea; coocu1214@knu.ac.kr (S.J.L.); sss@knu.ac.kr (S.S.); 2 Research Institute for Innovative Animal Science, Kyungpook National University, Sangju 37224, Republic of Korea; 3 Department of Animal Science, The Pennsylvania State University, University Park, PA 16802, USA

**Keywords:** citrinin, ferroptosis, *Euphorbia hypericifolia*, IPEC-J2, oxidative stress, tight junction, natural product

## Abstract

Citrinin (CTN), a mycotoxin commonly found in contaminated food and animal feed, impairs intestinal barrier integrity through oxidative stress and cytotoxicity. However, its link to ferroptosis, an iron-dependent form of regulated cell death, remains unclear. This study investigated whether CTN induces ferroptosis in intestinal epithelial cells and evaluated the protective role of *Euphorbia hypericifolia* (EH) against CTN-induced oxidative damage and tight junction (TJ) disruption. Using IPEC-J2 cells exposed to CTN, intracellular ferrous ion (Fe^2+^) levels, reactive oxygen species (ROS) accumulation, and TJ integrity were assessed using FerroOrange and DCFH-DA staining, RT-qPCR, immunofluorescence, and WST-1 assays. Additionally, a high-throughput screen of 459 natural products identified EH extract as a top candidate in mitigating CTN toxicity. The CTN treatment significantly elevated intracellular Fe^2+^ and ROS levels, downregulated antioxidant genes (notably *CAT*), and disrupted *ZO-1* expression and TJ morphology in IPEC-J2 cells, all hallmarks of ferroptosis-like cell death. Co-treatment with EH extract effectively reversed these effects, restoring antioxidant gene expression, reducing Fe^2+^ and ROS accumulation, and preserving TJ structure. Phytochemical profiling of EH extract revealed several bioactive compounds potentially responsible for its protective effects. These findings suggest that CTN induces ferroptosis-related cytotoxicity in IPEC-J2 cells, but EH alleviates this toxicity by modulating oxidative stress and iron homeostasis, supporting its potential use as a natural feed additive for intestinal protection

## 1. Introduction

Mycotoxins are toxic secondary metabolites produced by fungi. They commonly contaminate food and animal feed, posing significant risks to both animal and human health [1]. Among mycotoxins, aflatoxins, ochratoxins, fumonisins, deoxynivalenol, and zearalenone have been extensively studied for their detrimental effects on intestinal epithelial integrity [2,3,4,5]. These toxins induce oxidative stress, inflammation, and apoptosis and disrupt TJ proteins, ultimately impairing gut barrier function [6,7,8]. The mycotoxin citrinin (CTN) has attracted attention due to its widespread occurrence in contaminated grains and its ability to induce oxidative stress and cellular toxicity [9,10]. Although CTN is known to be harmful, its impact on intestinal barrier function has received less attention than that of other mycotoxins and further investigation is warranted.

Maintaining intestinal barrier integrity is crucial for nutrient absorption and host defense, with TJ proteins playing a pivotal role in regulating paracellular permeability [11,12]. However, exposure to mycotoxins such as CTN can compromise gut homeostasis by disrupting TJs [13]. CTN, a secondary metabolite produced by *Penicillium* and *Monascus* species, is known for its nephrotoxicity and hepatotoxicity, as well as its oxidative stress-inducing properties in intestinal epithelial cells [14,15]. Recent studies have suggested that oxidative stress-related cell death pathways contribute to CTN’s cytotoxicity, yet its specific relationship with ferroptosis remains unclear [16].

Distinct from apoptosis and necrosis, ferroptosis is an iron-dependent form of regulated cell death characterized by excessive lipid peroxidation and reactive ROS accumulation [17]. Unlike apoptosis, which is caspase-dependent and involves chromatin condensation and DNA fragmentation, ferroptosis is primarily driven by oxidative damage to membrane lipids [18]. A key feature of ferroptosis is the Fenton reaction, in which intracellular free iron catalyzes the conversion of H_2_O_2_ into highly reactive hydroxyl radicals, promoting lipid peroxidation and cell membrane destabilization [19]. In intestinal epithelium, ferroptosis has been implicated in inflammatory and oxidative stress-related conditions, suggesting a potential role in gut barrier dysfunction [20,21]. Given that CTN induces oxidative stress and lipid peroxidation, it is plausible that ferroptosis may contribute to its cytotoxic effects on intestinal epithelial cells [22]. However, further research is required to determine whether CTN directly triggers ferroptosis in this context.

Various dietary strategies to mitigate mycotoxin-induced intestinal damage, including natural feed additives, have been explored [23]. Natural compounds present in plant extracts, such as polyphenols and flavonoids, have shown promising antioxidant and anti-inflammatory properties, making them potential candidates for protecting the gut epithelium [24,25]. The genus Euphorbia encompasses a diverse range of medicinal plants with antioxidant, anti-inflammatory, and cytoprotective properties that show potential in mitigating oxidative stress and preserving epithelial integrity [26]. However, their role in alleviating mycotoxin-induced intestinal epithelial dysfunction remains largely unexplored, necessitating further investigation into their protective mechanisms.

This study evaluated the protective effects of EH extract against CTN-induced oxidative stress and TJ disruption in porcine enterocyte cells. By elucidating its potential mechanisms of action, we aim to provide a scientific basis for the development of EH extract as a natural feed additive to support intestinal barrier integrity and gut health.

## 2. Results

### 2.1. CTN Induces Intracellular Iron Accumulation

**Figure 1:**
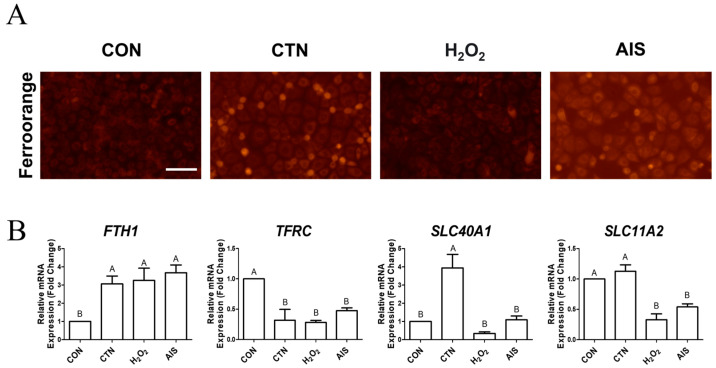
CTN induces intracellular iron accumulation in porcine intestinal epithelial cells. (**A**) Intracellular Fe^2+^ levels were detected using the FerroOrange fluorescent probe in untreated IPEC-J2 cells (CON) and IPEC-J2 cells treated with CTN, H_2_O_2_, or AIS. Increased fluorescence intensity indicates elevated Fe^2+^ accumulation, which was particularly strong in the CTN-treated group (scale bar = 100 μm). (**B**) The mRNA expression levels of the ferroptosis-related genes *FTH1, TFRC, SLC40A1*, and *SLC11A2* were analyzed using RT-qPCR in CON cells and CTN-, H_2_O_2_-, and AIS-treated cells. Data are presented as means ± SEs (*n* = 3). Statistical significance is denoted using letters; bars associated with different letters are significantly different (*p* < 0.05).

Previous studies have reported that the half-maximal inhibitory concentration (IC50) of CTN in IPEC-J2 cells is 160 μM [27]. Based on this, we used 160 μM CTN in our study to investigate its role in ferroptosis induction. To determine whether CTN induces intracellular iron accumulation, we performed cell staining using FerroOrange. A significant increase in iron ion fluorescence intensity was seen in both CTN-treated and ammonium iron(II) sulfate AIS-treated cells compared to the control group cells. However, no significant increase was observed in the H_2_O_2_-treatment group (Figure 1A), which suggests that CTN induces iron accumulation independently of oxidative stress. Additionally, mRNA expression levels of iron-related genes, including *FTH1*, *TFRC*, *SLC40A1*, and *SLC11A2*, were analyzed. The CTN treatment altered the expression of genes involved in iron storage and transport (Figure 1B). The differential expression patterns of iron-related genes observed in this study reflect a cellular response to citrinin-induced iron dysregulation. The upregulation of *FTH1* and *SLC40A1* suggests increased iron storage and export to mitigate intracellular iron accumulation. Conversely, the downregulation of *TFRC*, a key mediator of iron uptake, may represent a negative feedback mechanism to prevent further iron influx and protect cells from oxidative stress. These changes collectively indicate an adaptive response to maintain iron homeostasis under conditions of citrinin-induced ferroptotic stress.

### 2.2. CTN Induces the Production of Intracellular ROS

**Figure 2:**
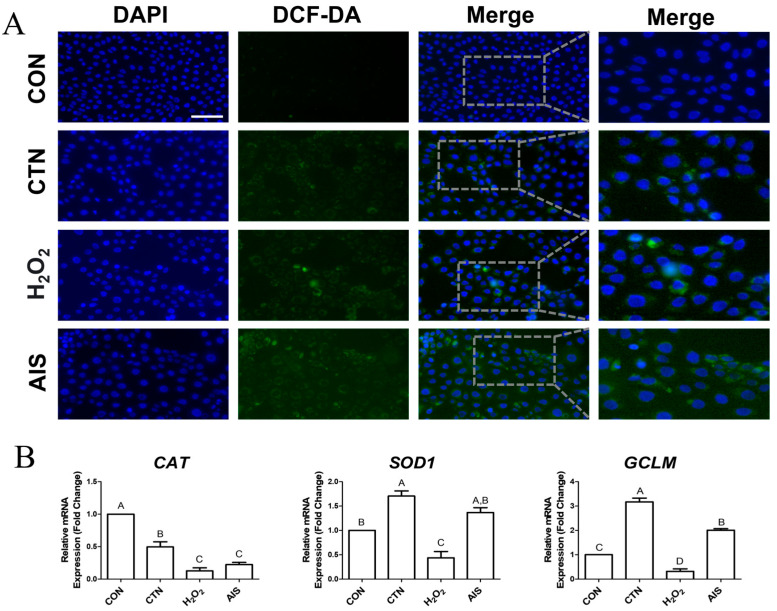
CTN induces ROS accumulation and alters antioxidant gene expression in IPEC-J2 cells. (**A**) Intracellular reactive ROS levels were assessed using the DCF-DA fluorescent probe in untreated IPEC-J2 cells (CON) and IPEC-J2 cells treated with CTN, H_2_O_2_, or AIS. Green fluorescence indicates ROS accumulation, and nuclei were counterstained with DAPI (blue) (scale bar = 100 μm). (**B**) The relative mRNA expression levels of the antioxidant-related genes *CAT*, *SOD1*, and *GCLM* were measured using RT-qPCR. Data are presented as the mean ± SE of three independent experiments (*n* = 3). Statistical significance is denoted using letters; bars associated with the same letter are not significantly different (*p* > 0.05).

To determine whether CTN induces ROS generation, we performed cell staining using DCFH-DA. Compared to the control group, significant increases in ROS levels were observed in the CTN-, AIS-, and H_2_O_2_-treated cells (Figure 2A). Furthermore, we analyzed the mRNA expression levels of the antioxidant-related genes *CAT*, *SOD1*, and *GCLM*, revealing that *CAT* expression was significantly lower in the CTN-treated cells than in the untreated cells, while *SOD1* and *GCLM* expression levels remained unchanged (Figure 2B). These findings suggest that CTN promotes intracellular ROS accumulation, potentially through the downregulation of CAT expression.

### 2.3. CTN Interferes with Tight Junctions

**Figure 3:**
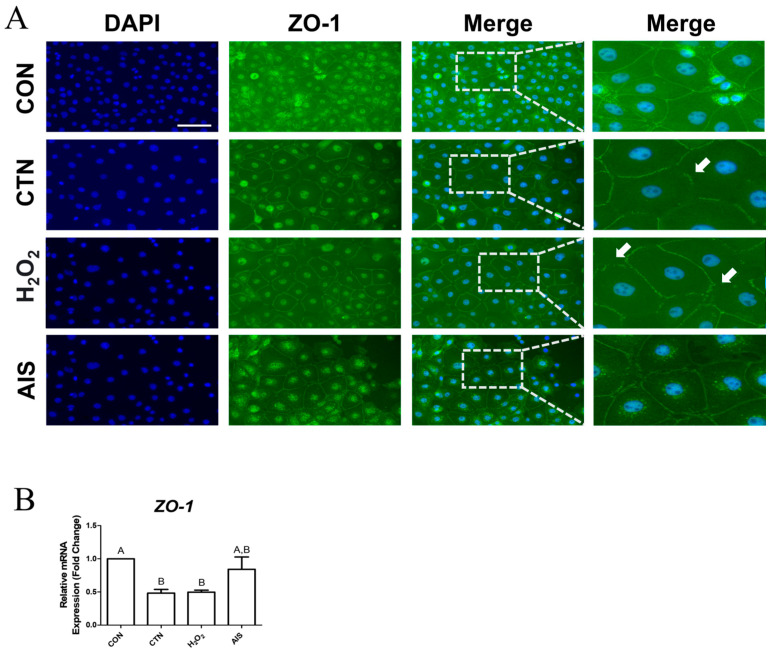
CTN disrupts tight junction integrity in IPEC-J2 cells. (**A**) Immunocytochemical staining of *ZO-1* in untreated IPEC-J2 cells (CON) and IPEC-J2 cells treated with CTN, H_2_O_2_, and AIS (scale bar = 100 μm). *ZO-1* was detected using an Alexa Fluor 488 secondary antibody (green), and nuclei were counterstained with DAPI (blue). The white arrows indicate damage to the TJs. (**B**) Relative mRNA expression of the tight junction gene *ZO-1* was quantified by RT-qPCR. Data are presented as means ± SEs (*n* = 3). Statistical significance is denoted using letters; bars associated with the same letter are not significantly different (*p* > 0.05).

To determine whether CTN disrupts TJs, we examined *ZO-1* expression using immunocytochemistry and RT-qPCR. Immunocytochemistry results showed that TJ structures were disrupted in the CTN-treated cells, and these disruptions were similar to those observed in the H_2_O_2_-treated cells (Figure 3A). Furthermore, RT-qPCR analysis revealed significantly lower *ZO-1* expression in both the CTN- and H_2_O_2_-treated cells than in control-group cells (Figure 3B). These findings suggest that CTN breaks down tight junctions between IPEC-J2 cells.

### 2.4. High-Throughput Screening of Natural Products to Assess Their Capacity to Alleviate CTN Toxicity

**Figure 4:**
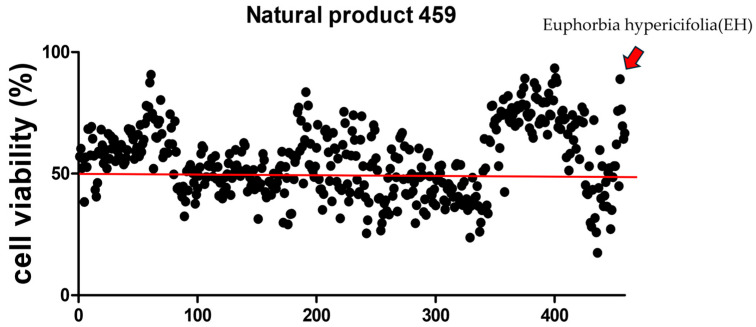
High-throughput screening for natural products mitigating citrinin toxicity. Cell viability was assessed using a WST-1 assay in IPEC-J2 cells treated with CTN at its IC50 value (160 μM) and 20 ng/μL of the respective 459 natural products for 24 h. The red line represents the cell viability of the CTN-only treatment group.

To evaluate whether any of the tested 459 natural products could mitigate CTN-induced cytotoxicity, IPEC-J2 cells were exposed to CTN in the presence of each natural product (Figure 4). Among the top five compounds that enhanced cell viability, EH extract was selected for further investigation. To further assess the protective effect of EH extract against CTN toxicity, IPEC-J2 cells were co-treated with CTN and EH for 24 h.

### 2.5. EH Alleviates CTN-Induced Intracellular Iron Accumulation

**Figure 5:**
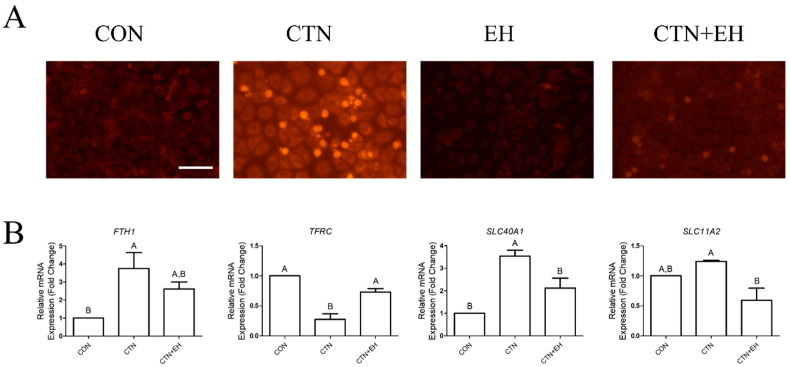
CTN induces intracellular iron accumulation in porcine intestinal epithelial cells. (**A**) Intracellular Fe^2+^ levels were detected using the FerroOrange fluorescent probe in untreated IPEC-J2 cells (CON) and IPEC-J2 cells treated with CTN, EH, or both (CTN+EH). Increased fluorescence intensity indicates elevated Fe^2+^ accumulation, which was particularly strong in the CTN-treated group (scale bar = 100 μm). (**B**) The mRNA expression levels of the ferroptosis-related genes *FTH1*, *TFRC*, *SLC40A1*, and *SLC11A2* were analyzed via qRT-PCR in CON cells and CTN-, EH-, and CTN+EH-treated cells. Data are presented as mean ± SE (*n* = 3). Statistical significance is denoted using letters; bars associated with the same letter are not significantly different (*p* > 0.05).

To investigate whether EH extract mitigates the toxicity of CTN, we performed cellular staining using FerroOrange to assess intracellular iron accumulation (Figure 5A). As previously observed, CTN treatment alone resulted in an increased fluorescence intensity, indicating enhanced iron accumulation. Interestingly, treatment with the EH extract alone did not produce any noticeable change in fluorescence intensity compared to the control, but co-treatment with CTN and EH extract led to a significant reduction in fluorescence intensity compared to CTN treatment, suggesting that EH alleviates CTN-induced intracellular iron accumulation. Additionally, we analyzed the mRNA expression levels of four iron-related genes, *FTH1*, *TFRC*, *SLC40A1*, and *SLC11A2* (Figure 5B). For *FTH1*, the co-treatment group exhibited no significant difference from the control, but the CTN treatment significantly increased expression. Additionally, the expression levels of *TFRC*, *SLC40A1*, and *SLC11A2* were significantly different in the co-treatment group compared to the CTN treatment alone, indicating that EH co-treatment suppresses CTN-induced changes in gene expression. Overall, EH extract demonstrates a clear ability to inhibit CTN-induced intracellular iron accumulation.

### 2.6. EH Alleviates the CTN-Induced ROS Accumulation

**Figure 6:**
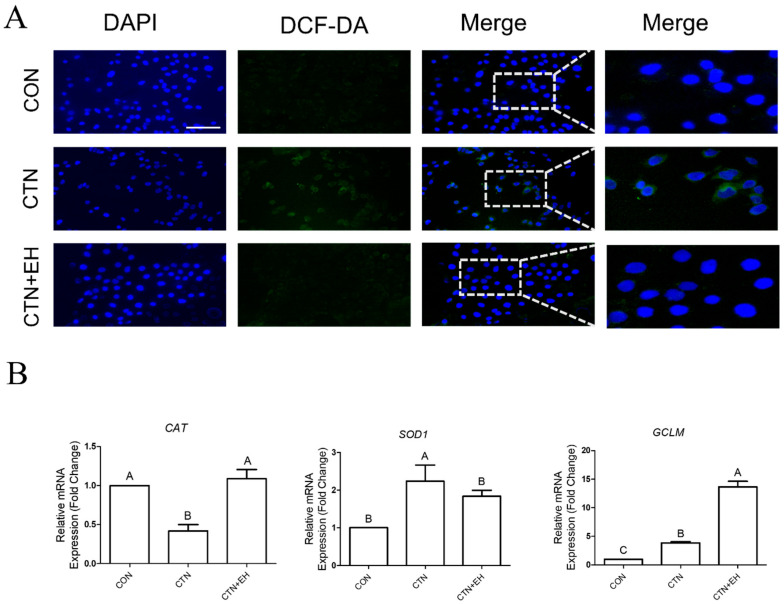
CTN induces ROS accumulation and alters antioxidant gene expression in IPEC-J2 cells. (**A**) Intracellular reactive ROS levels were assessed using the DCF-DA fluorescent probe in untreated cells (CON) and cells treated with CTN, EH, or both (CTN+EH). Green fluorescence indicates ROS accumulation, and nuclei were counterstained with DAPI (blue) (scale bar = 100 μm). (**B**) The relative mRNA expression levels of the antioxidant-related genes *CAT*, *SOD1*, and *GCLM* were measured using qRT-PCR. Data are presented as the mean ± SE of three independent experiments (*n* = 3). Statistical significance is denoted using letters; bars associated with different letters are significantly different (*p* < 0.05).

To determine whether EH extract suppresses CTN-induced ROS generation, we performed DCFH-DA cellular staining (Figure 6A). As previously observed, ROS levels were significantly increased in the CTN-treated cells. However, in the cells co-treated with CTN and EH, ROS levels were notably reduced, indicating a mitigating effect. We also analyzed the mRNA expression levels of the antioxidant-related genes *CAT*, *SOD1*, and *GCLM* (Figure 6B). The CTN treatment reduced *CAT* expression, and the co-treatment with EH restored the expression levels of *CAT* and *SOD1* to those comparable to the control group. Interestingly, *GCLM* expression was increased more in the co-treatment group than in the CTN-treatment group. These findings suggest that EH extract suppresses CTN-induced ROS accumulation, potentially through the regulation of antioxidant gene expression.

### 2.7. EH Alleviates the Disruption of Tight Junctions Induced by CTN

**Figure 7:**
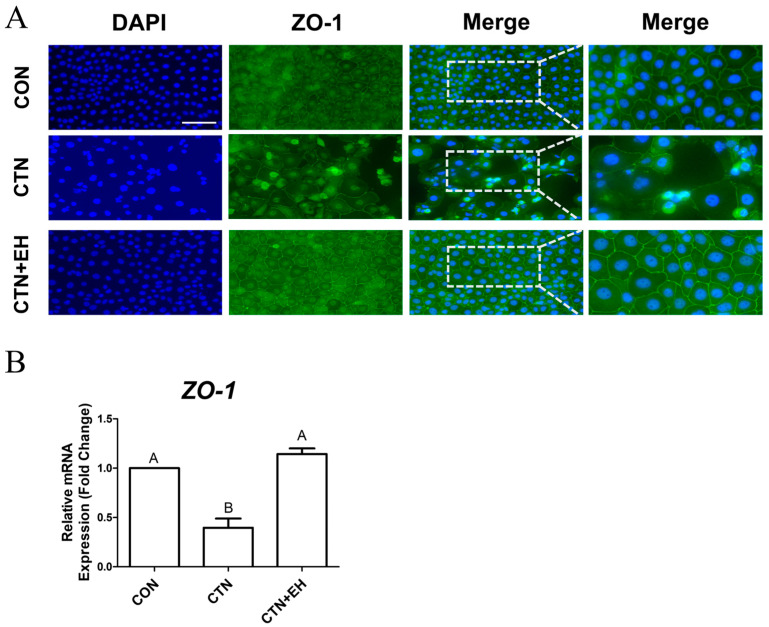
CTN disrupts tight junction integrity in IPEC-J2 cells. (**A**) Immunocytochemical staining of *ZO-1* in untreated cells and cells treated with CTN, EH, or both (CTN+EH). *ZO-1* was detected using an Alexa Fluor 488 secondary antibody (green), and nuclei were counterstained with DAPI (blue) (scale bar = 100 μm). (**B**) Relative mRNA expression of the tight junction gene *ZO-1* was quantified using qRT-PCR. Data are presented as means ± SEs (*n* = 3). Statistical significance is denoted using letters; bars associated with different letters are significantly different (*p* < 0.05).

To determine whether EH extract mitigates CTN-induced TJ disruption, we analyzed *ZO-1* expression using immunocytochemistry and RT-qPCR. Consistent with previous findings, immunocytochemical analysis revealed that the CTN treatment disrupted TJ structure (Figure 7A). However, this disruption was alleviated by the co-treatment with EH. Furthermore, the RT-qPCR analysis showed that, compared to the control, *ZO-1* expression was decreased in the CTN-treated group, and it was restored in the co-treatment group (Figure 7B). This indicates that EH plays a protective role against CTN-induced TJ damage in IPEC-J2 cells. Taken together, this study’s findings suggest that EH effectively mitigates CTN-induced cytotoxicity by regulating iron homeostasis, reducing ROS accumulation, and preserving tight junction integrity.

## 3. Discussion

This study is among the first to explore whether EH can mitigate CTN-induced ferroptosis and TJ disruption in intestinal epithelial cells. The results clearly demonstrate that EH alleviates CTN-induced cytotoxicity by regulating iron homeostasis, reducing intracellular ROS accumulation, and preserving TJ integrity. Citrinin is a well-documented mycotoxin known for its nephrotoxic, hepatotoxic, and oxidative properties [28,29,30]. Previous studies have highlighted the critical role of the *IP3R1*–*GRP75*–*VDAC1* complex in mediating ER–mitochondrial calcium transfer and mitochondrial dysfunction in response to CTN exposure, particularly in IPEC-J2 cells [31]. Disruption of this complex has been associated with increased mitochondrial ROS generation and epithelial barrier damage [32]. In addition, the mitochondrial electron transport chain (ETC) is another major source of ROS that may be dysregulated by CTN exposure [33]. Although our study did not directly assess the involvement of these organelle-specific pathways, the observed increase in ROS and the protective effect of EH suggest that these upstream signaling mechanisms may also be involved and warrant further investigation.

In this study, intracellular iron accumulation was significantly elevated in CTN-treated IPEC-J2 cells, as confirmed by FerroOrange staining. Furthermore, we compared the effects of exogenous H_2_O_2_ and AIS on intracellular iron levels to further investigate the mechanism of CTN-induced iron accumulation [34]. While AIS treatment, as expected, led to a marked increase in intracellular Fe^2+^ levels, H_2_O_2_ treatment did not cause such an increase. Interestingly, CTN treatment induced a comparable elevation in intracellular Fe^2+^ levels to that observed with AIS. These findings suggest that the CTN-induced accumulation of iron in IPEC-J2 cells is not merely a secondary effect of ROS elevation but may instead involve a distinct mechanism that disrupts iron homeostasis directly. This reinforces the hypothesis that CTN promotes ferroptosis through iron dysregulation in addition to oxidative stress. Additionally, CTN treatment led to distinct alterations in the expression of iron-related genes such as *FTH1*, *TFRC*, *SLC40A1*, and *SLC11A2*, suggesting a disruption of intracellular iron homeostasis and potential promotion of ferroptotic cell death. Specifically, *FTH1* was upregulated, indicating increased iron storage, while TFRC was consistently downregulated, likely reflecting a negative feedback response to limit iron uptake under oxidative stress. In contrast, *SLC11A2* expression was not significantly affected by CTN, unlike H_2_O_2_ and AIS treatments, implying that CTN may modulate iron import through a different regulatory mechanism. *SLC40A1* expression remained relatively stable, suggesting that iron export pathways are less responsive or subject to complex regulation depending on the type of oxidative insult. Furthermore, CTN markedly increased ROS levels and, among antioxidant-related genes, only *CAT* was significantly downregulated, indicating a compromised cellular capacity to eliminate H_2_O_2_ [35]. Catalase plays a crucial role in decomposing H_2_O_2_ into water and oxygen, which protects cells from oxidative stress, and the downregulation of CAT expression would likely facilitate the accumulation of H_2_O_2_ within cells [36]. The presence of free Fe^2+^ and H_2_O_2_ under such conditions can promote the Fenton reaction, wherein Fe^2+^ reacts with H_2_O_2_ to generate Fe^3+^, hydroxyl radicals (•OH), and hydroxide ions [37]. The hydroxyl radical is one of the most reactive ROS in biological systems and is capable of damaging lipids, proteins, and nucleic acids, thereby contributing to membrane lipid peroxidation and ferroptosis [38]. Taken together, our study’s findings suggest that CTN induces ferroptosis through a cascade involving reduced *CAT* expression, the accumulation of H_2_O_2_, enhanced intracellular Fe^2+^ levels, and activation of the Fenton reaction, ultimately leading to cellular damage and death.

**Figure 8:**
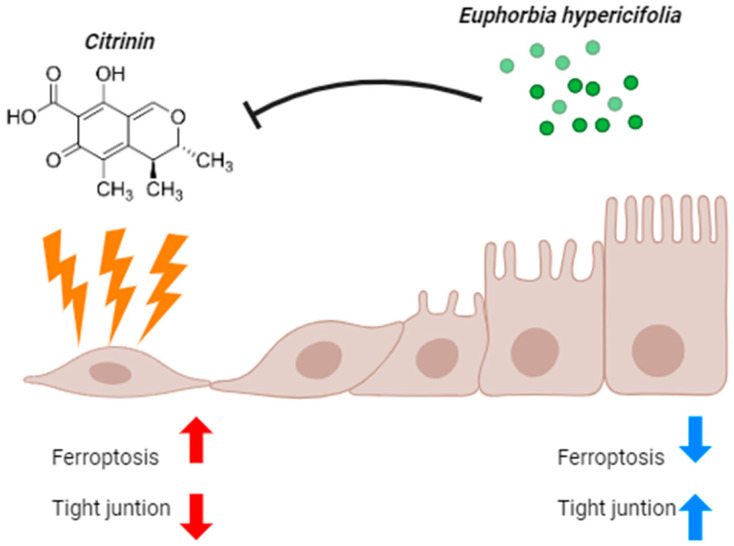
EH mitigates the ferroptosis and tight junction collapse caused by CTN.

Co-treatment with EH extract significantly reduced CTN-induced intracellular iron accumulation and ROS generation. Notably, the expression levels of *CAT* and *SOD1* were restored, and *GCLM* expression was further elevated over the levels seen in CTN-treated and untreated cells, suggesting that EH not only restores antioxidant defense mechanisms but also suppresses iron-driven oxidative stress by modulating iron metabolism [39]. Furthermore, the preservation of *ZO-1* expression and the maintenance of TJ structure in the CTN+EH co-treatment-group cells indicate that EH protects intestinal epithelial integrity by inhibiting ferroptosis and oxidative damage [40] (Figure 8).

**Figure 9:**
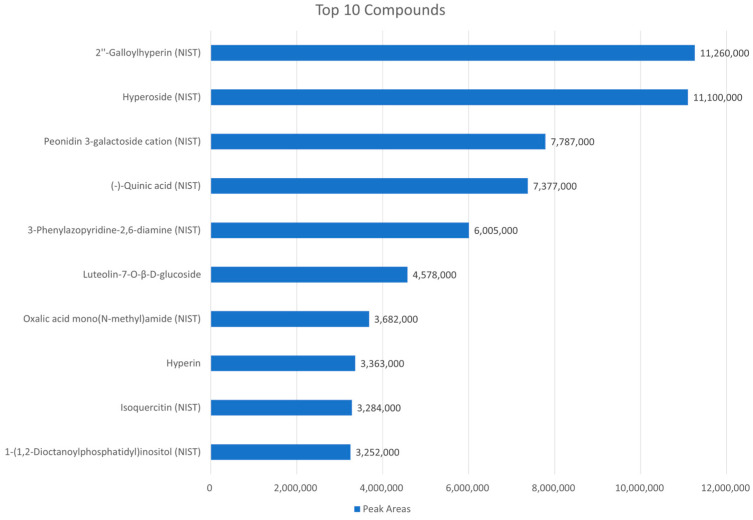
Top 10 major phytochemical compounds identified in *Euphorbia hypericifolia* extract by UHPLC-QTOF/MS analysis. The top 10 most abundant compounds identified in the *Euphorbia hypericifolia* extract (20 mg/mL) through qualitative analysis using UHPLC-QTOF/MS in both positive and negative ionization modes. The compounds were ranked in descending order based on their peak areas, and the bar lengths represent their relative abundances.

In our study, the UHPLC-QTOF/MS-based phytochemical profiling of the EH extract revealed the presence of several bioactive compounds, including 2″-galloylhyperin, hyperoside, peonidin 3-galactoside cation, and (−)-quinic acid (Figure 9). The compound 2″-galloylhyperin, a galloylated flavonoid derivative, is known for its potent free radical scavenging and lipid peroxidation-inhibiting capacities, which may directly counteract ferroptosis-associated oxidative damage [41]. Hyperoside, a flavonol glycoside, has been reported to scavenge ROS and enhance the expression of endogenous antioxidant enzymes, such as *CAT* and *SOD1*, aligning with our results [42]. Peonidin 3-galactoside cation, an anthocyanin compound, has previously been shown to regulate iron metabolism and inhibit ROS production through *Nrf2* activation, suggesting a role in modulating both upstream and downstream ferroptosis pathways [43]. In addition, (−)-Quinic acid is a key precursor in the biosynthesis of phenolic compounds and exhibits anti-inflammatory and antioxidant properties, which may contribute to maintaining epithelial integrity under oxidative stress [44]. The identification of these bioactive metabolites together in the EH extract provides a mechanistic rationale for its multifaceted protective effects. These compounds may act synergistically to suppress oxidative stress, regulate iron homeostasis, and preserve tight junction structure [42,45,46]. Further studies focusing on the isolation and functional analysis of each component will be valuable for elucidating their specific contributions to the anti-ferroptotic and barrier-protective actions of the extract. These compounds are known to possess strong antioxidant, anti-inflammatory, and iron-chelating activities, which may contribute to the observed protective effects against CTN-induced ferroptosis and tight junction damage. Despite the promising transcriptional responses observed in this study, an important limitation is the absence of in vivo validation. While mRNA expression analysis provides valuable initial insights into the potential mechanisms of EH extract, it does not necessarily translate to protein-level effects or physiological outcomes. Further studies utilizing in vivo models, particularly in mice or piglets, will be essential to confirm the intestinal protective effects and evaluate the systemic toxicity profile of EH extract. These models would enable a more comprehensive assessment of the extract’s efficacy and safety under physiological conditions, thereby facilitating its potential application in animal health or human therapeutics. To fully validate these findings and support translational application, further research is essential.

## 4. Materials and Methods

### 4.1. Cell Culturing

Cell line IPEC-J2 enterocyte cells (DSMZ, Braunschweig, Germany), originally isolated from piglet jejunal epithelium, were cultured in Dulbecco’s modified Eagle’s medium (DMEM) (Thermo Fisher Scientific, Wilmington, DE, USA) supplemented with fetal bovine serum at 10% and penicillin-streptomycin at 1% in a CO_2_ incubator.

### 4.2. RT-qPCR

Total RNA was extracted using the AccuPreP Universal RNA Extraction Kit (BioNEER, Daejeon, Republic of Korea), and RNA quantity and integrity were assessed using an Agilent 2100 Bioanalyzer (Agilent Technologies, Santa Clara, CA, USA) and a Thermo ND-2000 Spectrophotometer. Then, cDNA was synthesized from 1 μg of total RNA using the Dia-Star™ RT Kit (SolGent, Daejeon, Republic of Korea). Using the primers listed in Table 1, qPCR reactions were performed in triplicate in a Bio-Rad CFX96 Real-Time PCR Detection System with a protocol comprising an initial denaturation step at 95 °C for 3 min, followed by 40 cycles of 95 °C for 15 s, 56 °C for 15 s, and 72 °C for 15 s. Gene expression was normalized to that of GAPDH, and relative expression levels were calculated using the 2^−ΔΔCt^ method.

### 4.3. Measurement of Fe^2+^ Levels

The FerroOrange fluorescent probe was used to evaluate Fe^2+^ levels. First, IPEC-J2 cells were seeded at a density of 1 × 10^4^ cells per well in a 24-well plate and cultured for 24 h. The cells were then treated with CTN, H_2_O_2_, and EH extract for 24 h. After removing the culture medium, cells were washed twice with PBS and incubated in a serum-free medium containing 1 μmol/L of FerroOrange at 37 °C in the dark for 30 min. Fluorescence images were captured using fluorescence microscopy (Korea Labtech, Gyeonggi-Do, Republic of Korea).

### 4.4. Intracellular ROS Detection

IPEC-J2 cells were seeded at a density of 1 × 10^4^ cells per well in a 24-well plate and cultured for 24 h. Cells were then incubated overnight in DMEM, followed by treatment with CTN and EH extract for 24 h. After washing with PBS, cells were fixed and then stained in 10 μM DCF-DA (Cat. No. C400, Thermo Fisher Scientific) at 37 °C for 30 min. Experiments were performed in triplicate, and fluorescence images were obtained using fluorescence microscopy (Korea Labtech).

### 4.5. Immunofluorescence

After CTN treatment, IPEC-J2 cells grown on gelatin-coated glass coverslips were fixed with 4% paraformaldehyde. Cells were then incubated in a blocking solution, followed by an overnight incubation at 4 °C with an anti-ZO-1 antibody (1:200; Thermo Fisher Scientific) diluted in antibody buffer. The cells were subsequently washed and incubated for 1 h at room temperature in the dark with Alexa Fluor 488-conjugated anti-rabbit IgG secondary antibody. Lastly, cells were mounted using VECTASHIELD Antifade Mounting Medium with DAPI, and images were captured using fluorescence microscopy.

### 4.6. Cell Viability Assay

Cell viability was determined using the WST-1 assay (Roche Diagnostics GmbH, Mannheim, Germany). Cells were seeded at a density of 1 × 10^4^ cells per 100 μL in 96-well plates for 24 h and then incubated overnight in media. AIS (Sigma-Aldrich, St. Louis, MO, USA) was administered at concentrations of 25, 50, 100, 200, 300, 400, and 500 μM, and the plates were incubated for another 24 h. After a 2 h incubation with WST-1, absorbance at 450 nm was measured using a GloMax^®^ Discover Microplate Reader (Promega Corporation, Madison, WI, USA)

### 4.7. High-Throughput Screening

IPEC-J2 cells were seeded at a density of 1 × 10^4^ cells per 100 μL in 96-well plates and incubated overnight. Subsequently, 459 natural products (NPs), provided by the Nakdonggang National Institute of Biological Resources (Sangju, Republic of Korea), were individually administered at a concentration of 20 ng/μL in combination with 160 μmol/L citrinin (CTN), followed by a 24 h incubation. Among the NPs tested, Euphorbia hypericifolia extract was prepared by extracting dried aerial parts in 70% ethanol at a ratio of 1 g of sample to 60 mL of solvent. The mixture was subjected to ultrasonic-assisted extraction using a bath-type sonicator (SD-120H, Sejin Ultrasonics, Seoul, Republic of Korea) at 40 kHz and 180 W for 30 min, followed by 24 h incubation at room temperature. The extract was then filtered, concentrated under reduced pressure, lyophilized, and reconstituted in DMSO at a concentration of 20 mg/mL for experimental use. After a 2 h incubation with WST-1, cell viability was determined by measuring absorbance at 450 nm using a microplate reader.

### 4.8. Statistical Analysis

All experiments were conducted independently with three biological replicates (*n* = 3). Statistical significance was determined using the general linear model procedure (PROC-GLM) in SAS (v9.4). Cell viability data were analyzed using general linear regressions, while gene expression data were assessed using *t*-tests. Additionally, ANOVAs followed by Tukey’s multiple range tests were performed to compare differences among treatments. In all tests, *p* values < 0.05 were accepted as indicating statistical significance.

## 5. Conclusions

This study demonstrates that citrinin impairs intestinal epithelial barrier integrity via ferroptosis-related pathways characterized by excessive iron accumulation, ROS generation, and tight junction disruption. Co-treatment with EH extract significantly attenuates these effects by restoring antioxidant defense systems and modulating iron metabolism. These protective effects are likely attributed to the presence of flavonoids and phenolic acids in EH that act synergistically to counteract oxidative stress. Collectively, our findings suggest that EH has potential as a functional natural additive for maintaining intestinal barrier integrity in livestock. Nonetheless, further in vivo studies and detailed molecular analyses of ferroptosis-specific markers are warranted to confirm its efficacy and clarify the underlying mechanisms

## Figures and Tables

**Table 1:** List of PCR primers.

Target Gene	Description	Accession No.		Sequence (5′–3′)
*FTH1*	Ferritin heavy chain 1	XM_005660803.3	Forward	AAC AGT GCT TGG ACG GAA C
			Reverse	AAG TGG GGG TCA TTT TTG TC
*TFRC*	Transferrin receptor	NM_214001.1	Forward	GCT TAT TTT GGG CAG ACC TC
			Reverse	TCA CCG AGT TTT CAG CAT TG
*SLC40A1*	Solute carrier family 40 member 1	XM_003483701.4	Forward	TCA TTG GCT GTG GTT TCA TT
			Reverse	CAA GGG TTT TGG CTC AGT TT
*SLC11A2*	Solute carrier family 11 member 2	NM_001128440.1	Forward	CCA CCA CAT ACA ACA CCA CA
			Reverse	CAC TTG AGC ATC CAA CAT CG
*SOD1*	Superoxide dismutase 1	NM_001190422	Forward	GAG GGA ATG TTT ACT GGG TGA
			Reverse	GCA CGC AAA TAA AAC TGC TC
*CAT*	Catalase	NM_214301	Forward	GGC TTT TGG CTA CTT TGA GG
			Reverse	AGG GTC ACG AAC TGT GTC AG
*GCLM*	Glutamate-cysteine ligase modifier subunit	XM_001926378	Forward	CTT GCC TCT TGC TGT GTG AT
			Reverse	CGA TGT CAG GGA TGC TTT C
*ZO-1*	Zonula occludens 1	XM_021098856	Forward	GAT CCT GAC CCG GTG TCT GA
			Reverse	TTG GTG GGT TTG GTG GGT TG
*GABDH*	Glyceraldehyde-3-phosphate dehydrogenase	NM_001206359	Forward	ACA CCG AGC ATC TCC TGA CT
			Reverse	GAC GAG GCA GGT CTC CCT AA

## Data Availability

All data pertinent to the study’s results are contained within the article.

## Abbreviations

The following abbreviations are used in this manuscript:

CTN	Citrinin
EH	*Euphorbia hypericifolia*
TJ	Tight junction
ROS	Reactive Oxygen Species
Fe^2+^	Ferrous Ion
DCF-DA	2′,7′-Dichlorodihydrofluorescein Diacetate
IPEC-J2	Intestinal Porcine Epithelial Cells-J2
H_2_O_2_	hydrogen peroxide
SIA	ammonium iron(II) sulfate
